# Active surveillance of thyroid microcarcinomas

**DOI:** 10.20945/2359-3997000000181

**Published:** 2019-10-03

**Authors:** Claudio R. Cernea

**Affiliations:** 1 Departamento de Cirurgia Faculdade de Medicina Universidade de São Paulo São Paulo SP Brasil Departamento de Cirurgia , Faculdade de Medicina da Universidade de São Paulo , São Paulo , SP , Brasil

There is a recent trend in the thyroid cancer literature towards a more conservative approach to papillary microcarcinomas of the thyroid gland. The widespread acceptance of lobectomy as adequate treatment for most patients is part of this tendency. Undoubtedly, the most conservative attitude concerning these indolent cancers is the active surveillance, pioneered by Miyauchi and Ito in Japan (1-7) and already being used in other centers around the world (8,9). In the present issue of *Archives of Endocrinology and Metabolism* , there are two excellent South American manuscripts addressing the approach to papillary carcinomas of the thyroid gland. I would like to congratulate the authors of both papers for their important contribution to the already existing controversy concerning the adoption of active surveillance for patients with these very small and usually (but not invariably) indolent cancers.

Based upon the formidable amount of data coming from the experience of University of Kuma, it is possible to delineate the possible candidates to active surveillance:

1. Patients with age ≥ 60 years-old;
2. Bethesda VI, with no cytological evidence of high-risk variants of papillary thyroid cancer;
3. Tumors ≤ 1.0 cm;
4. Tumors located in the center of the thyroid lobe, far from the tracheoesophageal groove and from the thyroid capsule (even with no evidence of extrathyroidal extension);
5. No clinical or radiological evidence of lymph node metastasis or distant metastasis;
6. No previous exposure to neck irradiation and
7. Medical contraindications for surgery.

Just for the sake of discussion, it is noteworthy that there was no emphasis on the role of molecular factors associated with more aggressiveness, like TERT mRNA expression (10). Evidently, the possibility to positively identify at FNAB, molecular/genetic factors associated with aggressive thyroid papillary microcarcinomas, thus supporting the adoption of a more aggressive approach at the initial evaluation, is still in its infancy.

Rosario and cols. suggests in their manuscript, published in this issue of *Archives of Endocrinology and Metabolism* , that most patients with cytologically proven thyroid papillary microcarcinomas should undergo active surveillance, highlighting the excellent prognosis of those subjects who will eventually undergosurgery, provided that growth, lymph node metastasis or extrathyroidal extension will be detected (11). However, we must keep in mind that Brazil is a country with continental dimensions, with 210 million inhabitants. Moreover, the public health assistance is the only option for more than 78% of the population (12). Unfortunately, the quality of, literally, a lifelong active surveillance in such a gigantic health system is inevitably rather heterogeneous and often problematic, especially on less-developed areas of the country. In real life we, Head and Neck Surgeons, often face troubles to get a close follow-up for much more aggressive cancers, as oral and hypopharyngeal carcinomas, sometimes impairing an effective salvage treatment of a tumor recurrence. Therefore, instead of using the expression “active surveillance”, I would rather suggest “effective surveillance”, sadly not always available in Latin America.

There is another aspect that deserves a comment. The only available large-scale, prolonged follow-up series of patients with thyroid microcarcinomas submitted to active surveillance is the Kuma Hospital’s one. Obviously, there are several features of this population which are not universally applicable. Among them, I would like to emphasize the biopsychosocial differences, meaning that the acceptance rate of the active surveillance by patients in a Latin American country would probably be quite different from Japanese individuals. Surprisingly, Ito et al. recently reported that, even at Kuma Hospital, it took around 20 years to obtain a significant rate of acceptance of the active surveillance (13). They observed a slow increase in the acceptance rate from 30% in the period from 1993 to 1997, to 88% from 2014 to 2016.

In this issue of *Archives of Endocrinology and Metabolism* , Smulever and Pitoia also reported their remarkable experience with the proposal of active surveillance for a consecutive series of 136 subjects with thyroid papillary microcarcinomas (14). Just 34 (25%) of the patients actually accepted the active surveillance. In fact, nearly one-third of these 34 patients subsequently chose to undergo surgical treatment claiming anxiety. Interestingly enough, most subjects who decided to be operated on, preferred to undergo a total thyroidectomy, despite the fact that the initial proposal of the surgical team was lobectomy in many instances. I suspect that these numbers would probably be easily reproducible in other Latin American countries, and I really hope that, in the future, similar reports in other geographic areas will appear in the literature as well, in order to offer a more global picture. At the conclusion of their abstract, the authors state that: *Although not easily accepted in our cohort of patients, active surveillance would be safe and easily applicable in highly experienced centers* .

Finally, I would like to quote a statement made by Zanocco and cols. in a Clinical Update recently published at *JAMA Insights* (15), with which I fully agree: *While active surveillance of small intrathyroidal thyroid cancers has the potential to reduce morbidity from surgical treatment, adoption of this strategy in the United States is in the early stages, and it remains unknown whether the favorable outcomes reported by specialized centers will be widely reproducible. Hopefully, future advances in molecular diagnostic testing of fine needle aspiration material will aid patient selection by identifying the minority of thyroid cancers that will aggressively grow and spread without surgical excision. Until then, standardized mechanisms for evaluating individual preferences and risk tolerance for different treatment-related complications are needed to help patients decide between immediate surgery and observation of low-risk thyroid cancer.*
